# Strength and Power Responses to a Power-Oriented Resistance Training Model across an Entire Soccer Competitive Season

**DOI:** 10.5114/jhk/218820

**Published:** 2026-04-02

**Authors:** Renan F. H. Nunes, Lucas A. Pereira, Juan P. Fiorenza, Carlos A. Paes, Rafael Grazioli, Piotr Bryk, Irineu Loturco

**Affiliations:** 1 Health and Performance Department, Criciúma Esporte Clube, Criciúma, Brazil.; 2 NAR—Nucleus of High Performance in Sport, São Paulo, Brazil.; 3 Department of Human Movement Sciences, Federal University of São Paulo, São Paulo, Brazil.; 4 Health and Performance Department, Coritiba Foot Ball Club, Curitiba, Brazil.; 5 Department of Physical Education, São Francisco University, Bragança Paulista, Brazil.; 6 Institute of Sport Sciences, Jerzy Kukuczka Academy of Physical Education in Katowice, Katowice, Poland.; 7 Scientific Department, São Paulo Football Federation, São Paulo, Brazil.; 8 FSI—Football Science Institute, Granada, Spain.

**Keywords:** strength training, muscle strength, team sports, athletic performance

## Abstract

This study investigated longitudinal changes in strength- and power-related performance across an entire competitive season in elite soccer players using a frequent and systematic monitoring approach. Twenty-three professional players competing in the Brazilian first division were monitored over seven consecutive months. Athletes performed two to three resistance training (RT) sessions per week, with one weekly “control” session used to adjust training loads and assess neuromuscular status through bar-derived measures obtained in the half-squat exercise. Relative power (RP), relative strength (RS), and estimated one-repetition maximum (1RM) were recorded across 28 training sessions using the load associated with maximum power output. Weekly variations were analyzed using a 4-week rolling average, and pre-, mid-, and post-season periods were compared using repeated-measures ANOVA. Across the season, RP, RS, and 1RM exhibited a gradual and consistent positive trend. While only isolated meaningful changes were detected when individual sessions were compared with rolling averages, significant improvements were observed at the post-season time point compared with both pre- and mid-season values (p < 0.05). Notably, despite a modest ~5% increase from pre- to post-testing, absolute training loads did not change significantly across the season, indicating that performance gains were achieved without meaningful increases in training load magnitude. These findings demonstrate that a power-oriented RT model, supported by continuous monitoring and load adjustments, can effectively preserve and enhance strength and power throughout a competitive soccer season. This approach appears to mitigate commonly reported in-season declines in neuromuscular performance and offers a practical strategy for managing RT under congested competitive schedules.

## Introduction

In soccer, particularly during long competitive seasons, players are chronically exposed to high volumes of sport-specific training (e.g., small-sided games and technical-tactical sessions) and official matches, which are predominantly aerobic-based in their physiological demands (Redkva et al., 2018; Stølen et al., 2005). It is well established that these activities may interfere with the effective development and maintenance of neuromuscular qualities such as maximum strength and power (Loturco et al., 2015b; Silva et al., 2015). This phenomenon is commonly referred to as the “concurrent training effect” and represents a practical challenge for soccer coaching staff (Loturco et al., 2015b; Silva et al., 2015). An additional barrier frequently faced by practitioners involves the constraints imposed by congested fixture schedules in modern soccer, which often limit—or even preclude—the implementation of traditional resistance training strategies throughout the season (Cormack et al., 2008; Issurin, 2010; Morgans et al., 2014).

Strength and power capacities are strongly associated with multiple performance-related attributes, including acceleration and sprinting abilities, jumping performance, rapid changes of direction and cutting maneuvers (Freitas et al., 2019b; Suchomel et al., 2016). Consequently, reductions in strength and power levels during the competitive phase may negatively affect players’ performance and potentially increase the risk of musculoskeletal and non-contact injuries (França et al., 2024; Suchomel et al., 2016). However, maintaining sufficient strength- and power-oriented stimuli during the competitive season is somewhat difficult in contemporary soccer, as sport-specific training sessions and match demands frequently take precedence over resistance training, and congested schedules, travel demands, and the required time for recovery between matches further restrict the implementation of complementary training activities (Morgans et al., 2014; Silva et al., 2015).

To address these constraints, coaches and practitioners have increasingly adopted alternative resistance training strategies characterized by lower training volumes (i.e., a reduced number of exercises, sets, and repetitions) and the use of light-to-moderate loads, typically performed with maximal intended movement velocity. In this context, training approaches aimed at maximizing power output (i.e., training at the optimum power zone) (Loturco et al., 2016; 2013), rather than increasing training volume or intensity (e.g., using higher percentages of one-repetition maximum [1RM]), have been proposed as a more feasible and effective training method during the competitive season (Cormier et al., 2024). Recent reviews and studies (Cormier et al., 2024; Freitas et al., 2019a; Loturco et al., 2015b) suggest that power-oriented resistance training may be effective not only in enhancing performance, but also in preserving strength and power levels in team sports when training time and recovery resources are limited.

Despite these practical recommendations, few longitudinal studies have examined the effects of such training strategies across an entire competitive season using frequent and systematic monitoring of strength and power output and adjustments of training loads, especially in professional soccer players. Most available studies rely on short intervention periods (e.g., 4–6-week pre-season), limited assessment time points, or insufficient control of training load, which restricts our understanding of how these neuromuscular qualities evolve under real-world competitive conditions (Cormier et al., 2024; Freitas et al., 2019a; Loturco et al., 2015b). In particular, there is a lack of studies tracking weekly variations in neuromuscular performance over extended periods (e.g., >6 months) while accounting for the cumulative effects of sport-specific training and competition.

Therefore, the purpose of this study was to investigate whether meaningful changes in strength- and power-related performance occur throughout a long competitive season in elite soccer players. Specifically, we aimed to monitor weekly variations in strength- and power-derived metrics (i.e., relative strength [RS], relative power [RP], and maximum strength) over seven consecutive months to determine whether these capacities were maintained, improved, or deteriorated during the competitive soccer season. By adopting a systematic, longitudinal monitoring approach, this study aims to provide a clearer understanding of the real-world responses of neuromuscular performance in elite soccer players exposed to prolonged competitive demands.

## Methods

### Participants

Twenty-three elite soccer players (age: 24.3 ± 4.2 years; age range: 18.6–32.8 years; body height: 1.51 ± 0.67 m; body mass [BM]: 79.5 ± 8.0 kg) from the same professional soccer club participated in this study. The initial dataset comprised 29 athletes, all of whom had a minimum of 5–6 years of resistance training experience as part of their professional training background. Nevertheless, only those who attended at least 90% of the total resistance training sessions over the monitoring period were included in the final analyses. Players who missed assessments due to injury or absence and did not meet this attendance criterion were excluded from the analyses. Accordingly, all analyses were conducted using a per-protocol approach, and no data imputation was performed. The final sample included 2 goalkeepers and 21 outfield players, distributed as follows: 6 defensive players (central defenders and full-backs), 8 midfielders, and 7 forwards. The athletes were members of a professional soccer club competing in the first division of the Brazilian National Championship, the most important soccer competition in Brazil. The study was approved by the Ethics Committee of the Federal University of São Paulo, São Paulo, Brazil (protocol number: 7.748.149; approval date: 06 August 2025), and all participants provided written informed consent prior to participation.

### Study Design

This descriptive longitudinal study analyzed the seasonal variations in strength and power performance of elite soccer players over a 7-month period. The typical training schedule of the athletes during the study period is depicted in Table 1. Soccer players participated in two to three resistance training sessions per week. The first resistance training session of the week was used as a “control” session to assess and adjust the load in the half-squat (HS) exercise. During this session, neuromuscular status was monitored through the assessment of peak power (PP), mean propulsive velocity (MPV), and the load associated with maximal power production, enabling the detection of week-to-week variations in RS and RP performance. These measures were subsequently used to individually adjust training loads, ensuring that players consistently trained within their optimum power zone. Based on negative or positive variations in PP, players performed, respectively, 4 sets of 4 repetitions or 6 sets of 6 repetitions of the HS, with training volume adjusted according to individual decreases or increases in HS power, replicating power-oriented training schemes already used in previous studies conducted over shorter periods (i.e., periods ≤6 weeks) (Freitas et al., 2019a; Loturco et al., 2015b, 2016). Such variations (i.e., decreases or increases ≥5%) in PP values were also used as thresholds to determine the completion of the HS power test (Loturco et al., 2022). Regardless of the number of sets and repetitions performed, a minimum rest interval of 2–3 min was provided between HS trials to ensure complete athletes’ recovery and to maintain high movement velocities throughout the entire training session (Cormie et al., 2011; González-Badillo et al., 2011). During all resistance training sessions, athletes were asked to move the bar as fast as possible in each repetition, thereby ensuring that the highest possible magnitude of force was applied against the given load. Over the 7-month period, data from 28 HS training sessions were continuously monitored. Prior to each training session, players performed a standardized warm-up that included general exercises (e.g., 10 min of moderate-paced running followed by 3 min of dynamic lower-limb stretching) and submaximal squat attempts. Besides HS training, in order to avoid potential confounding factors related to our main outcomes, any other loaded exercises involving triple extension (i.e., concomitant extension of ankles, knees, and hips) were not prescribed. Hence, in addition to HS training, players executed only core exercises (e.g., front plank, side plank, and stability-ball rollouts) and unloaded exercises such as Nordic hamstring and Copenhagen adduction exercises.

**Table 1:** Typical weekly training schedule during the study period.

Monday	Tuesday	Wednesday	Thursday	Friday	Saturday	Sunday
Recovery session	*RT session (*load control) TEC-TAC	SSG (45–60’) or official match	RT session TEC-TAC or recovery session	TEC-TAC (60–90’) #CT session	SSG (30’)	Official match

Note: During weeks with only one official match, players typically completed three RT sessions. RT: resistance training; TEC: technical training; TAC: tactical training; SSG: small-sided games; CT: core training + unloaded training sessions, usually performed on Fridays (≈ 80% of the time)

### Bar-Derived Output in the Half-Squat Exercise

Bar-derived PP and MPV were assessed in the HS exercise using the load corresponding to maximum power production (Loturco et al., 2015a, 2015b, 2016) during 28 resistance training sessions. The test was conducted on a Smith machine (Hammer Strength Equipment, Rosemont, IL, USA). Players completed three repetitions at maximal intended velocity, with 15-s intervals between attempts. A linear velocity transducer (Vitruve Encoder, Madrid, Spain), sampling at 100 Hz, was attached to the bar to measure and record mechanical variables until a decrease of at least 5% in PP was observed (Loturco et al., 2022). The highest PP output obtained was subsequently divided by the players’ BM and utilized for individual analysis of the changes in RP production (Loturco et al., 2024b). Based on the MPV values, 1RM was determined using a validated formula (Martínez-Cava et al., 2019). Lastly, the RS measure was obtained by dividing the absolute 1RM load by the players’ BM (Loturco et al., 2024b).

### Statistical Analysis

Data are presented as means, standard deviation (SD), and confidence intervals (CI). Normality of the data was checked and confirmed using the Shapiro-Wilk test and by inspection of Q-Q plots. Based on the variables collected, a 4-week rolling average along with the smallest worthwhile change (i.e., SD × 0.2) (Hopkins, 2004) were calculated and used as the comparison variable for the subsequent 4 weeks. Meaningful changes were noted when the lower bound of the CI did not include zero (i.e., the average value of the last 4-week period) (Fethney, 2010). In addition, variables obtained in sessions 1–4, 13–16, and 25–28 (defined as pre, middle, and post, respectively) were grouped and compared using a repeated-measures analysis of variance (ANOVA). To determine the magnitude of the differences between pre-, middle-, and post-intervals, effect sizes (ES) were calculated and interpreted using the thresholds proposed by Rhea (2004) for highly trained subjects, as follows: <0.25, 0.25–0.50, 0.50–1.00, and >1.00 for trivial, small, moderate, and large effect sizes, respectively. The level of significance was set at *p* < 0.05. Given the exploratory nature of the study and the strong interrelationships among the analyzed variables, no formal adjustment for multiple comparisons was applied (Rothman, 1990). Thus, results were interpreted based on effect sizes, confidence intervals, and practical relevance rather than on *p*-values alone (Hopkins, 1997, 2004).

## Results

**Figure 1:**
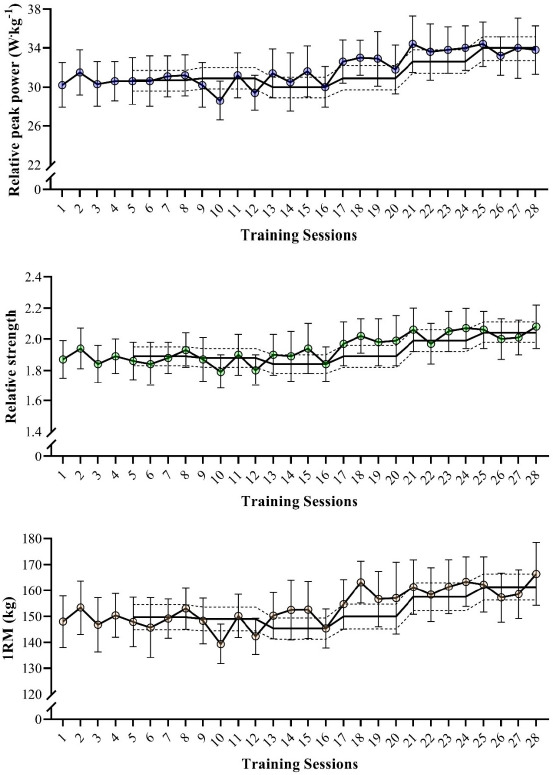
Variations in the different variables assessed across the 28 controlled training sessions. 1RM: one-repetition maximum in the half-squat exercise. Data are presented as means and 95% confidence intervals. Continuous lines represent the rolling average and dotted lines represent the smallest worthwhile change

**Figure 2:**
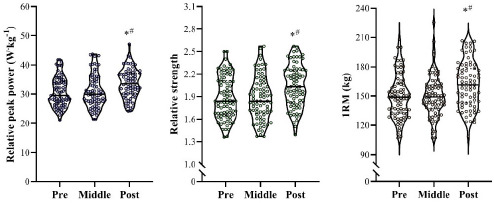
Changes in the different variables when comparing the pre-, middle-, and post-periods. 1RM: one-repetition maximum in the half-squat exercise. * p < 0.05 vs. pre; # p < 0.05 vs. middle

Figure 1 shows the variations in the different variables assessed over the 28 controlled training sessions. In general, a constant and gradual increase was observed for PP, RS, and 1RM across the 7-month period. Nevertheless, only isolated meaningful changes were identified when comparing individual session values with the 4-week rolling average. In Figure 2, changes in the different variables obtained when comparing the pre, middle, and post periods are shown. Significant increases were noted for PP, RS, and 1RM at post (*p* < 0.05) compared with the pre and middle intervals (ES [95% CI] = 0.58 [0.26; 0.90] and 0.48 [0.16; 0.80] for PP; 0.54 [0.22; 0.85] and 0.55 [0.23; 0.87] for RS; 0.52 [0.20; 0.83] and 0.53 [0.21; 0.85] for 1RM, when compared with pre and middle, respectively). Finally, despite a modest ~5% increase from pre- to post-testing, no significant changes were observed in the absolute loads used during the resistance training sessions when comparing the three different periods (pre = 92 ± 9.8 kg; middle = 91.2 ± 9.4 kg; post = 95.8 ± 8.7 kg; *p* > 0.05; ES varying between 0.19 [−0.50; 0.12] and 0.38 [0.06; 0.70]).

## Discussion

The primary aim of this study was to examine longitudinal changes in strength- and power-related performance across an entire competitive season in elite soccer players using a frequent and systematic load monitoring approach. The main findings indicate that, over seven consecutive months and 28 monitored training sessions, RP, RS, and maximum strength (1RM) displayed a gradual and consistent positive trend. However, despite this progressive pattern, only a limited number of changes were classified as meaningful when individual sessions were compared against the corresponding 4-week rolling averages (i.e., the first four weeks of monitoring). Importantly, although progressive increases were observed throughout the season, statistically significant improvements were detected only at the post-season time point, compared with both pre- and mid-season periods. This distinction highlights that statistical significance emerged primarily at the end of the season, whereas adaptations occurred more gradually over time. Collectively, these findings suggest that the training model adopted during the competitive season was sufficient not only to preserve neuromuscular performance, but also to promote practically relevant gains in strength and power by the end of the season.

From a longitudinal perspective, the gradual increase observed in the three performance variables (i.e., RP, RS, 1RM) contrasts with commonly reported seasonal patterns in team sports, in which strength and power capacities tend to remain unchanged or deteriorate during congested competitive phases (Mohr et al., 2005; Silva et al., 2018). Previous studies in soccer and other team sports frequently report reductions or stability in neuromuscular performance across the season (Cormack et al., 2008; Freitas et al., 2021; Loturco et al., 2024a), often attributed to high volumes of aerobic-based training, cumulative match fatigue, and limited opportunities to adopt structured resistance training methods (Loturco et al., 2015b; Mohr et al., 2005; Reilly et al., 2009). In this context, the present findings suggest that the proposed training model may be more effective when applied across prolonged competitive periods characterized by high match density and limited recovery time. The absence of performance decline across the season—and the presence of a progressive positive trend—represents a notable and somewhat unexpected outcome. These findings indicate that, despite the well-known constraints imposed by the competitive calendar, neuromuscular performance can be effectively maintained — and even improved — when resistance training is appropriately designed and individualized (i.e., executed using individually calculated loads).

An important aspect of the current results is the distinction between progressive trends and meaningful changes. Although RP, RS, and 1RM increased consistently across the season, only isolated changes exceeded the threshold for meaningfulness when compared with the rolling 4-week baseline. This reinforces the need to distinguish between statistical significance and practical relevance (Bishop et al., 2021; Hopkins, 2004), especially in elite sport contexts. Such a pattern is likely related to the conservative analytical approach adopted, which aimed to detect robust changes beyond normal week-to-week variability rather than short-term fluctuations. This is not unexpected in high-performance sport settings, where neuromuscular adaptations during the competitive season typically occur gradually and are influenced by the need to balance match performance, training, and recovery demands (Freitas et al., 2021; Loturco et al., 2015b; Mohr et al., 2005; Pereira et al., 2022). Consequently, the lack of frequent meaningful changes should not be interpreted as an absence of adaptation, but rather as evidence of stable progression under real-world competitive conditions (i.e., a national soccer championship).

The most notable finding of the study was the significant improvement observed at the post-season time point for all neuromuscular variables. This result is particularly relevant because it contrasts with the natural tendency for strength and power to decline toward the end of the season in many team sports (Cormack et al., 2008; Lehnert et al., 2014). Specifically in soccer, late-season reductions in neuromuscular performance are commonly reported and are attributed to accumulated fatigue, reduced training stimuli, limited recovery time, and injury-related issues (Freitas et al., 2021; Lehnert et al., 2014; Loturco et al., 2015b; Pereira et al., 2022). Our findings suggest that the proposed training model may be beneficial for players exposed to prolonged competitive demands and cumulative seasonal fatigue, as players not only avoided these declines but also achieved meaningful improvements in RP, RS, and maximum strength (Loturco et al., 2022). This outcome indicates that the resistance training model implemented throughout the season was effective in mitigating the negative effects typically associated with concurrent training effects and prolonged competitive exposure (Freitas et al., 2021; Loturco et al., 2015b; Silva et al., 2015).

A plausible explanation for these positive adaptations lies in the weekly adjustment of training loads based on bar-power output measures. By regularly identifying the loads associated with maximal power production (Loturco et al., 2015a, 2015b, 2016), players were consistently exposed to training stimuli that were individualized and aligned with their neuromuscular status. This strategy probably facilitated favorable neuromuscular adaptations by maintaining training intensity within an “optimum” and moderate loading range (i.e., 45–60% 1RM) (Loturco et al., 2013, 2022), even in the presence of seasonal fatigue accumulation and fluctuations in the match load (Loturco et al., 2013, 2016, 2022). Training at or near the optimum power zone (Loturco et al., 2013, 2016, 2022), likely provided an effective neuromuscular stimulus while minimizing excessive physiological and metabolic stress, which is essential during congested fixture schedules (Cormier et al., 2024; Loturco et al., 2022). In this sense, the systematic load-adjustment strategy adopted in the present study may represent a key mechanism underpinning the observed maintenance and late-season improvements in strength and power performance.

It is also important to contextualize these findings within the comprehensive literature on team-sport performance across competitive seasons. A common trend of neuromuscular performance stability, characterized by minimal gains or decreases during the competitive season, has been reported not only in soccer but also in other team sports such as rugby and basketball (Loturco et al., 2015b, 2024a; Petway et al., 2020). The current results extend this evidence by suggesting that power-oriented resistance training models can be effective for athletes with prior exposure to structured strength-power training (i.e., professional soccer players). When resistance training is carefully managed and continuously monitored through the use of moderate loading conditions (i.e., loads moved at approximately 1 m·s⁻^1^, capable of maximizing power output) (Cormier et al., 2024; Loturco et al., 2015a, 2022), it is possible to move beyond simple maintenance and achieve meaningful improvements by the end of the season (Cormier et al., 2024; Loturco et al., 2022). This reinforces the practical value of longitudinal monitoring strategies coupled with the use of light-to-moderate loads executed with maximal intended movement velocity, supporting the feasibility and effectiveness of power-oriented resistance training as an in-season approach (Cormier et al., 2024; Loturco et al., 2015b, 2024b; Mcbride et al., 2002).

This study is inherently limited by its descriptive longitudinal design without randomization and without a control group, which precludes causal inference. Nonetheless, the applied nature of the monitoring strategy, the extended duration of the observation period (i.e., seven months), and the high frequency of load measurements enhance the ecological validity of the findings. Additionally, the use of a rolling average approach allowed for a more detailed interpretation of performance changes, which better reflects real-world practice compared with traditional pre-post experimental designs. Another important limitation is that match exposure (i.e., minutes played) and key indicators of soccer training load (e.g., sRPE, heart rate-based load, or GPS-derived metrics) were not monitored or controlled due to inherent constraints related to resources, equipment availability, and logistics. Moreover, factors such as squad rotation, unequal match exposure among soccer players, variations in weekly microcycle structure, and injury occurrence or player availability were not controlled and may have influenced the magnitude and timing of the observed adaptations. Thus, the potential influence of match load and sport-specific training demands on the observed neuromuscular adaptations cannot be fully isolated and should be considered when interpreting the present findings. Future studies should aim to replicate these findings in different competitive and training contexts using more controlled designs and further explore how similar monitoring and training strategies may influence injury risk, match performance, and long-term athlete development.

## Conclusions and Practical Implications

This study demonstrates that a power-oriented resistance training model, supported by frequent and systematic monitoring and weekly load adjustments, can effectively preserve (and ultimately enhance) strength and power capacities throughout a long competitive soccer season. Over seven consecutive months, elite players exposed to substantial match and training demands not only avoided the commonly reported seasonal declines in neuromuscular performance but also achieved significant improvements by the end of the season. These findings challenge the commonly held assumption that strength and power inevitably stabilize or deteriorate during congested competitive schedules and reinforce the practical relevance of implementing well-designed training strategies under real-world conditions (Freitas et al., 2021; Loturco et al., 2015b, 2024a; Mohr et al., 2005; Silva et al., 2015). Our results highlight the importance of prioritizing training quality over volume during the competitive season. The consistent use of loads associated with maximal power production, combined with maximal intended movement velocity, appears to provide a sufficient neuromuscular stimulus while minimizing excessive mechanical and metabolic stress. Moreover, the weekly adjustment of loads based on bar-power output allowed training prescriptions to remain aligned with players’ neuromuscular status, which may be essential in environments characterized by frequent matches, travel demands, and limited recovery time. The gradual nature of the observed adaptations underscores that meaningful strength-power improvements during the season are likely to occur over prolonged periods rather than through abrupt changes. As such, continuous monitoring approaches may offer practitioners a more realistic and sensitive framework for interpreting performance evolution than traditional and widely used pre-post assessments. Collectively, these findings support the use of individualized, power-oriented resistance training and continuous monitoring as an effective strategy for maintaining and improving neuromuscular performance in elite soccer players throughout the annual training season.
